# Molecular Assessment of Chelonid Alphaherpesvirus 5 Infection in Tumor-Free Green (*Chelonia mydas*) and Loggerhead (*Caretta caretta*) Sea Turtles in North Carolina, USA, 2015–2019

**DOI:** 10.3390/ani10111964

**Published:** 2020-10-25

**Authors:** Annie Page-Karjian, Maria E. Serrano, Jeffrey Cartzendafner, Ashley Morgan, Branson W. Ritchie, Christopher R. Gregory, Joanne Braun McNeill, Justin R. Perrault, Emily F. Christiansen, Craig A. Harms

**Affiliations:** 1 Harbor Branch Oceanographic Institute, Florida Atlantic University, Fort Pierce, FL 33431, USA; jcartzendafner@gmail.com (J.C.); aschenk2019@fau.edu (A.M.); 2 Center for Marine Science and Technology, North Carolina State University, Morehead City, NC 27695, USA; meserran@ncsu.edu (M.E.S.); emily.christiansen@ncaquariums.com (E.F.C.); caharms@ncsu.edu (C.A.H.); 3 Infectious Disease Laboratory, University of Georgia, Athens, GA 30602, USA; britchie@uga.edu (B.W.R.); crg@uga.edu (C.R.G.); 4 National Oceanic & Atmospheric Administration, National Marine Fisheries Service, Southeast Fisheries Science Center, Beaufort, NC 27954, USA; jbmcneill@yahoo.com; 5 Loggerhead Marinelife Center, Juno Beach, FL 33408, USA; jperrault@marinelife.org; 6 North Carolina Aquariums, Raleigh, NC 27954, USA

**Keywords:** antibodies, ChHV5, ELISA, qPCR, rehabilitation, subclinical infection

## Abstract

**Simple Summary:**

Fibropapillomatosis is a debilitating tumor disease of sea turtles that is sometimes fatal. This disease is a key concern for sea turtle rehabilitation facilities due to its infectious nature, as it is associated with a virus called chelonid alphaherpesvirus 5. This is the first study to analyze antibodies to this virus in loggerhead sea turtles and represents the most complete dataset on viral detection in sea turtles encountered in the more northern latitudes of their habitat in the western Atlantic.

**Abstract:**

Fibropapillomatosis is associated with chelonid alphaherpesvirus 5 (ChHV5) and tumor formation in sea turtles. We collected blood samples from 113 green (*Chelonia mydas*) and 112 loggerhead (*Caretta caretta*) turtles without fibropapillomatosis, including 46 free-ranging turtles (20 green turtles, 26 loggerheads), captured in Core Sound, North Carolina, and 179 turtles (93 green turtles, 86 loggerheads) in rehabilitative care in North Carolina. Blood samples were analyzed for ChHV5 DNA using quantitative polymerase chain reaction (qPCR), and for antibodies to ChHV5 peptides using an enzyme-linked immunosorbent assay (ELISA). None of the samples from foraging turtles tested positive for ChHV5 by qPCR; ELISA was not used for foraging turtles. Samples from 18/179 (10.1%) rehabilitating turtles tested positive for ChHV5 using qPCR, and 32/56 (57.1%) rehabilitating turtles tested positive for antibodies to ChHV5 using ELISA. Five turtles that tested positive by qPCR or ELISA at admission converted to being undetectable during rehabilitation, and five that initially tested negative converted to being positive. Both sea turtle species were significantly more likely to test positive for ChHV5 using ELISA than with qPCR (*p* < 0.001). There was no difference in the proportions of green turtles versus loggerheads that tested positive for ChHV5 using qPCR, but loggerheads were significantly more likely than green turtles to test positive for ChHV5 using ELISA. This finding suggests that loggerheads infected with ChHV5 at some point in their life may be more able than green turtles to mount an effective immune response against recrudescent infection, pointing to species-specific genetic differences in the two species’ immune response to ChHV5 infection. This is the first study to analyze antibodies to ChHV5 in loggerhead turtles and represents the most complete dataset on ChHV5 DNA detection in sea turtles encountered in the more northern latitudes of their western Atlantic habitat.

## 1. Introduction

Sea turtles inhabiting the coastal habitats of the southeastern United States face many threats to their health and survival, including the infectious, tumor-causing disease fibropapillomatosis, hereafter referred to as “FP” [1]. This disease is most commonly reported in green turtles (*Chelonia mydas*), but it has been sporadically described in all seven sea turtle species, including loggerhead sea turtles (*Caretta caretta*; hereafter, loggerheads) [2,3,4,5,6,7,8]. FP is a major concern in sea turtle rehabilitation facilities, since it is transmitted horizontally and necessitates special quarantine protocols [9]. When present, FP may have accompanying detrimental clinical signs such as anemia and/or opportunistic coinfections, can extend rehabilitation times, and can complicate prognoses [10,11,12]. While FP prevalence is high (>50%) in some areas of the southeastern United States (e.g., some parts of Florida), it is much less frequently reported in the more northern parts of this region [7,13,14,15].

FP is associated with chelonid alphaherpesvirus 5 (ChHV5), which is consistently identified in fibropapilloma tumors and was shown to be a requirement for tumor growth in a series of infectivity trials and molecular tests [16,17,18,19,20]. ChHV5 is shed from infected epithelial cells on and adjacent to fibropapilloma tumors and possibly in urine, and it may persist in water column for hours to days [16,19,21,22,23,24]. Therefore, ChHV5 infection and/or FP development is a risk factor for turtles entering rehabilitation facilities, particularly those facilities that admit and treat turtles with FP [12]. Identification of subclinical ChHV5 infections could help to determine the quarantine status and reduce transmission opportunities among rehabilitating turtles and could provide information about the pathobiology of ChHV5 in a wildlife rehabilitation setting [22,25]. Diagnosing ChHV5 infections in the absence of external fibropapilloma tumors can be accomplished using molecular techniques including polymerase chain reaction (PCR), which can be used to detect circulating ChHV5 DNA in blood samples from turtles with active infections, and serology, which can be used to detect circulating antibodies in blood samples from turtles that were infected at some point in their immunologically detectable past [22,26,27]. In general, herpesviruses persist in an infected host for life, forming a latent infection with periodic viral shedding during periods of stress such as concomitant disease, malnutrition, temperature changes (high or low), movement of animals, introduction into an established collection, or breeding activity [28,29,30,31,32,33]. Because ChHV5 infections are likely lifelong, the detection of either viral nucleic acids or antibodies to previous infections are both interpreted to suggest ChHV5 infection [27].

Among sea turtle populations in the southeastern United States, North Carolina-based aggregations are relatively under-studied with regards to FP and ChHV5 prevalence. Based on stranding data, FP is historically thought to be less common in the more northern sea turtle populations including North Carolina [7,13,34]. In-water trawl sampling conducted in South Carolina and Georgia during the early 2000s revealed that only 2 of 946 (0.2%) captured loggerheads had FP (diagnosis confirmed via histopathology) [35]. In-water pound net sampling in Core and Pamlico Sounds, North Carolina during 2004–2007 resulted in 205 captured loggerhead turtles, but FP was not observed in any of them [14]. It is possible, however, that ChHV5 infections may be spreading and becoming more prevalent in northern foraging juvenile sea turtles. With this study, we assess foraging and rehabilitating sea turtle populations in North Carolina using physical examination and molecular and serological techniques to provide data on FP and ChHV5 infection rates for green and loggerhead sea turtles.

## 2. Materials and Methods 

### 2.1. Animals and Blood Samples

The free-ranging portion of the study population consisted of foraging green and loggerhead turtles caught in Core Sound, North Carolina by pound net and entanglement net during in-water population assessments conducted by the United States National Marine Fisheries Service (NMFS) during 2016, 2018 and 2019. The captive portion of the study population consisted of juvenile green turtles and juvenile-to-adult loggerhead turtles admitted to sea turtle rehabilitation centers in North Carolina, USA during 2015–2019 (Karen Beasley Sea Turtle Rescue and Rehabilitation Center, Sea Turtle Assistance and Rehabilitation Center at the North Carolina Aquarium on Roanoke Island). Turtles were identified by external flipper tags and/or internal passive integrated transponder (PIT) tags. Complete external examinations were performed on all turtles, including assessments for fibropapilloma tumors. 

Blood samples (up to 3 mL) were collected from the dorsal cervical sinus of all study turtles using an appropriately sized BD Vacutainer^®^ blood collection system (Becton−Dickinson, Franklin Lakes, NJ, USA) or heparinized needle and syringe, and aseptic technique. A single blood sample was collected from free-ranging turtles, while multiple (up to seven throughout rehabilitation) blood samples were collected from many rehabilitating turtles, including upon admission and just prior to release. The packed cell volume (PCV, %) was determined by placing whole blood samples into microhematocrit tubes and centrifuging for 5 min at 14,800 *g* (12,000 rpm), and it was interpreted using a hematocrit microcapillary tube reader. The plasma total solid concentrations (g/L) were estimated using the heparinized plasma from the centrifuged microhematocrit tubes and a handheld refractometer. All plasma samples had a hemolysis score of 0 or <1+ [36]. Whole blood samples were placed into 1 mL tubes coated with the anticoagulant ethylenediaminetetraacetic acid (EDTA) and into 1 mL lithium heparin-coated tubes. Lithium heparin tubes were centrifuged at 5000 *g* for 5 min, and plasma aliquots were pipetted into sterile 2 mL cryogenic vials. Because plasma samples were also used for biochemistry profiles in some turtles for medical diagnostic purposes, and because some blood samples were not of an adequate volume to produce both whole blood and plasma, there were more whole blood samples than plasma samples available for analysis. EDTA tubes and cryogenic vials were stored at −80 °C for up to three months and then shipped on dry ice to laboratories at FAU Harbor Branch (Fort Pierce, FL, USA) and University of Georgia (Athens, GA, USA) for a molecular analysis. 

### 2.2. DNA Extraction and qPCR

At FAU Harbor Branch, genomic DNA (gDNA) was extracted from whole blood samples stored in EDTA tubes using the DNeasy Blood and Tissue Kit (Qiagen, Germantown, MD, USA). The resultant gDNA samples were assessed for the ChHV5 UL30 gene segment using a singleplex, probe-based quantitative polymerase chain reaction (qPCR) assay and the methodologies described in detail by Page−Karjian et al. [22]. By following best qPCR practices, as described in the MIQE Guidelines, the assay parameters were optimized and potential pitfalls related to assay contamination were prevented [37]. All positive qPCR results were confirmed via Sanger sequencing (Genewiz, South Plainfield, NJ, USA) and alignment to ChHV5 sequences in the GenBank database. 

### 2.3. ELISA Detection of Antibodies to ChHV5 Peptide

Separated plasma samples were analyzed for antibodies to ChHV5 peptides at the University of Georgia Infectious Disease Laboratory (UGA IDL) in Athens, Georgia USA. Prior to the sample analysis, the plasma samples were noted to contain gelatinous clots and/or flecks of fibrin and were therefore presumed to be adequate for assays validated in-house for turtle serum. To evaluate them for infection by ChHV5 in a turtle’s immunologically detectable past, samples were analyzed in triplicate using an enzyme-linked immunosorbent assay (ELISA) that tests for antibodies to a ChHV5 purified synthetic peptide antigen. This assay was developed and validated based on modifications of previously published protocols [26,38] and was performed using the laboratory’s standard operating procedures with negative and positive control sera. The peptide HerbstFibropapGlyh4 referenced in Herbst et al. [26] (CKALKSGKIEGEDRK, New England Peptide, Gardner, MA, USA) was used as the antigen. Positive serum controls for ChHV5 were obtained from turtles confirmed by histopathology to be positive for FP. Negative serum controls were obtained from <7-month-old (posthatchling) green turtles from a captive population hatched and reared at the Cayman Turtle Centre with no history of confirmed FP. These controls are standards developed by the UGA IDL for use in the described commercially available assays; they were not developed specifically for this project. 

### 2.4. Statistical Analysis

The mean and standard deviation (SD) were calculated for the number of days between samples for rehabilitating turtles with multiple samples, and for the PCV and plasma total solid values for rehabilitating green and loggerhead turtles (at admission and prerelease). The N–1 chi-squared test was used to test for differences between proportions; for green turtles, loggerheads and both species combined, we compared: (1) turtles that tested positive for ChHV5 via qPCR versus ELISA; (2) free-ranging versus rehabilitating turtles that tested positive for ChHV5 via qPCR; and (3) turtles that tested positive for ChHV5 via qPCR and ELISA upon entry into a rehabilitation facility versus as established patients. The N–1 chi-squared test was also used to test for differences between proportions of green turtles versus loggerheads that tested positive for ChHV5 via qPCR and ELISA [39,40,41]. Shapiro−Wilk tests indicated that the data were not normally distributed, so nonparametric tests were selected to compare the variable median values. Specifically, Mann−Whitney U-tests for unpaired data were used to analyze for differences in the median ChHV5 DNA copy number, PCV, and plasma total solids concentration between green and loggerhead turtles that tested positive for the virus via qPCR. Cohen’s Kappa (κ) coefficient was calculated to determine the qualitative level of agreement between the qPCR and ELISA assays for ChHV5 [42]. Statistical analyses were conducted using MedCalc for Windows, version 19.2.0 (MedCalc Software, Ostend, Belgium) with a statistical significance set at α = 0.05. 

### 2.5. Ethics Statement

The sample and data collection and use were conducted by authorized personnel under North Carolina Wildlife Resources Commission Endangered Species Permits 15ST44, 16ST42, 17ST42, 18ST42, 19ST42, and 20ST42; National Marine Fisheries Service permits #16733 and #21233; and North Carolina State University Institutional Animal Care and Use Committee protocols 17-044-O (approved 2017) and 20-155-O (approved 2020). Sample use by Dr. Page−Karjian was approved by the Florida Fish & Wildlife Conservation Commission under Marine Turtle Permit #139, and by the Florida Atlantic University Institutional Animal Care and Use Committee under Animal Product Use protocol #A(T)16-02 (approved 2016).

## 3. Results

### 3.1. Animals and Blood Samples

A total of 338 blood samples were analyzed from 225 turtles, including 113 juvenile green turtles (23.1–44.4 cm SCL_max_) and 112 large juvenile (43.2–74.7 cm SCL_max_) and adult (75.1–101.9 cm SCL_max_) loggerheads (Table 1). These size classes are typical of turtles that have recruited back to their neritic habitat in the southeastern United States [43,44]. Blood samples were analyzed from 46 free-ranging sea turtles collected during population assessments in Core Sound during May and October 2016 (*n* = 29); October, November, and December 2018 (*n* = 14); and June 2019 (*n* = 3). From the turtles admitted to rehabilitation centers, single samples were collected from 117 turtles at or shortly after admission, and a total of 174 paired (≥2) samples were collected from 62 turtles (Table 1) from May 2015 through June 2019. The reasons for admission to rehabilitation included chronic debilitation, cold stunning, buoyancy disorder, boat strike, hook ingestion, fishing line or rope entanglement, trauma from dredge interaction, and trauma of unknown origin. None of the turtles included in the study had external tumors consistent with fibropapillomatosis. For 93 green turtles at the time of rehabilitation admission, the mean ± SD PCV was 30 ± 8%, and the mean ± SD plasma total solids concentration was 29 ± 14 g/L. For 41 rehabilitating green turtles for which multiple samples were collected, the mean ± SD PCV and the mean ± SD plasma total solids concentration at the prerelease physical examination were 30 ± 4% and 58 ± 9 g/L, respectively. For 80 loggerheads, the mean ± SD PCV and the mean ± SD plasma total solids concentration at the time of admission into rehabilitation were 27 ± 9% and 41 ± 14 g/L, respectively. For 19 rehabilitating loggerheads for which multiple samples were collected and the PCV and total solids data were available, the mean ± SD PCV and the mean ± SD plasma total solids concentration at the prerelease physical examination were 29 ± 6% and 51 ± 13 g/L, respectively.

### 3.2. Molecular Detection of ChHV5 DNA and Antibodies

The detailed qualitative results for samples tested with the qPCR and ELISA assays are shown in Table 2. The raw data are available online within the Open Science Framework data repository at https://osf.io/9zm7g/. Plasma samples were not available from the free-ranging turtles; therefore, those animals were not tested using ELISA. Overall, samples from 18 rehabilitating turtles (eight green turtles, 10 loggerheads) tested positive for ChHV5 using qPCR, and there were 32 rehabilitating turtles (six green turtles, 26 loggerheads) with at least one sample that tested positive for antibodies to ChHV5 using ELISA. The results of the chi-squared tests are presented in Table 3. Cohen’s Kappa coefficient statistical analysis revealed no agreement between qPCR and ELISA results in green turtles and a fair agreement between the two assays in loggerheads (Table 4).

For the 62 turtles undergoing rehabilitation that had more than one sample analyzed over time, the mean ± SD number of days between samples was 71 ± 49 (range: 8–250). Of these 62 turtles, three (4.8%) had a qPCR-positive sample at admission, including one green turtle and two loggerheads; all three of those turtles had one or more subsequent qPCR-negative sample(s), within an average of 54 ± 5 days (range: 50–59 days). Fourteen turtles (22.6%) had an ELISA-positive sample at admission, including one green turtle and 13 loggerheads; of those, three loggerheads had one or more subsequent ELISA-negative samples within an average of 59 ± 34 days (range: 28–96 days). One of those loggerheads (1.6%) had a blood sample that tested positive for ChHV5 via both qPCR and ELISA at admission, with two subsequent samples that tested negative via both assays. Three loggerheads (4.8%) entered rehabilitation with a qPCR-negative blood sample and subsequently had a qPCR+ sample after an average of 133 ± 77 days (range: 86–221 days) in captive care. One loggerhead (1.6%) initially had an ELISA-negative sample and subsequently had an ELISA-positive sample 33 days later, while another loggerhead had three serial plasma samples that were ELISA-negative, then positive after 55 days, then negative again after another 41 days.

No turtles had more than one blood sample that tested positive for ChHV5 via qPCR, while 54 turtles (87.1%), including 40 green turtles and 14 loggerheads, had multiple samples (up to seven) that consistently tested negative for ChHV5 via qPCR. There were nine turtles (14.5%), including two green turtles and seven loggerheads, with multiple samples (up to six) over time that consistently tested positive for ChHV5 via ELISA. Six turtles (9.7%), including five green turtles and one loggerhead, had multiple (up to three) samples over time that consistently tested negative for ChHV5 via ELISA.

The copy number data for the 18 blood samples that tested positive for ChHV5 DNA via qPCR are summarized in Table 5. All qPCR products that tested positive for ChHV5 DNA were confirmed via Sanger sequencing, with ≥95% identity with available ChHV5 partial genome sequences (i.e., GenBank accession number HQ878327.2). A Mann–Whitney U-test indicated that the median ChHV5 DNA copy number was significantly higher in blood samples from loggerheads than in green turtle samples (*p* = 0.02, 95% CI = 17.6–652.2, *U* = 66). There was also a much wider range in the ChHV5 DNA copy number in loggerheads (50.4–8017 copies) than in green turtles (50.4–254 copies). Neither the median PCV nor plasma total solids concentration significantly differed between green turtles and loggerheads that did and did not test positive for ChHV5 via qPCR or ELISA (all were *p* > 0.05).

## 4. Discussion

Due to its infectious and debilitating nature and a lack of complete information about transmission routes, FP remains an important concern in sea turtles, particularly in a rehabilitation or captive care setting [12]. With this study, we provide molecular diagnostic data to lend insight into the current and previous ChHV5 infection status in free-ranging and rehabilitating green and loggerhead sea turtles in the southeastern United States. None of the free-ranging turtles evaluated in this study tested positive for ChHV5 via qPCR, and they were not evaluated using ELISA. Samples from rehabilitating turtles, however, tested positive for ChHV5 via both qPCR and ELISA, including turtles that tested positive at admission to rehabilitation. This suggests that turtles admitted to rehabilitation can have active or previous, yet subclinical, ChHV5 infections that resolve over time and with supportive care. This finding may be related to immunosuppression associated with their cause(s) of stranding, such as chronic debilitation, cold stunning, boat strike wounds, fishing gear entanglement, or other various reasons that sea turtles strand [45,46,47,48]. These data also suggest that subclinical ChHV5 infections are not uncommon in stranded and rehabilitating green and loggerhead turtles.

The majority of published reports on FP cases and the detection of ChHV5 DNA and antibodies from the east coast of the United States are centered around green turtles in the more southern latitudes, particularly Florida [13,22,26,39,49], although there are sporadic case reports of FP in sea turtles as far north as North Carolina [7]. To date, this study represents the most complete dataset on ChHV5 DNA detection in sea turtles encountered in the more northern latitudes of their habitat in the western Atlantic. Since none of the turtles evaluated in this study had external tumors consistent with FP, positive qPCR results suggest active, yet subclinical ChHV5 infections resulting in ChHV5 DNA-emia [22]. Spontaneous FP tumor regression has been documented in free-ranging green turtles [50]; however, it is unknown whether any of the turtles in this study had previous FP tumors that regressed prior to sampling. ChHV5 DNA has been previously detected in blood and skin samples taken from free-ranging and rehabilitating green and loggerhead turtles without FP [22,25,51]. Such subclinical infections may represent early ChHV5 infections prior to tumor development or previously latent infections that recrudesce during times of stress, including clinical illness and stranding [52]. For the 18 turtles that tested positive for ChHV5 via qPCR, the mean viral copy number (698 ± 1888 viral copies per μg DNA; range = 50−8067) was similar to ChHV5 DNA copy numbers previously reported for whole blood samples taken from juvenile green turtles (3300−28,000 copies of viral DNA per µL) without external FP [22].

Based on the results of the chi-squared tests (Table 3), both green and loggerhead turtles were significantly more likely to test positive for ChHV5 antibodies using ELISA than for viral DNA with qPCR. These serological data demonstrate that ELISA-positive turtles had circulating antibodies to ChHV5 at the time of sampling, suggesting that they were infected with ChHV5 in their immunologically detectable past. Serology is a more sensitive method than qPCR for detecting previous viral infection and associated immune responses, since it does not depend on current infections and circulating nucleic acids resulting from viral replication. Other studies have demonstrated a high ChHV5 antibody seroprevalence in wild green turtles with and without FP [26,27,53]. For example, in Hawaii 20–40% of green turtles were seropositive for ChHV5, whereas in Florida green turtles were uniformly seropositive, including up to 60% of turtles without FP [53]. Another study on free-ranging green turtles inhabiting the east coast of Florida found 80.0–87.3% seroprevalence in turtles without FP, versus 88.2–100% seroprevalence in turtles with tumors [26]. Notably, in that study, plasma samples from eight juvenile loggerheads were also seropositive for ChHV5, despite only one of those turtles having tumors consistent with FP [26]. Such data demonstrate that ChHV5 is endemic in free-ranging turtles along the east coast of Florida, while the present study demonstrates ChHV5 endemicity in the northern extent of these populations’ range on the eastern seaboard of the United States. Although the gH peptide target antigens used in the ELISA presented here were similar to those used in a previous study in which assay cross-reactivity between ChHV5 and a related herpesvirus (chelonid alphaherpesvirus 6) was ruled out, the possibility of other antigenically similar herpesviruses being present in wild populations cannot be excluded [26,27]. The absent-to-fair level of agreement found here between the two diagnostic tests is not surprising, since they test for different things (active infection versus antibodies). Thus, each test provides incomplete information about the status of herpesvirus infection, and the most comprehensive diagnostic information can be obtained when the two assays are applied in tandem [27]. Future studies using paired qPCR and ELISA assays for ChHV5 on free-ranging sea turtles, particularly loggerheads, would provide an important comparison to the data presented here on rehabilitating turtles.

There was no difference in the proportions of green turtles versus loggerheads that tested positive for ChHV5 using qPCR; however, loggerheads were significantly more likely than green turtles to test positive for ChHV5 using ELISA. This finding suggests that loggerheads infected with ChHV5 at some point in their life may be more able than green turtles to mount an effective immune response against recrudescent infection, pointing to species-specific genetic differences in the two species’ immune response to ChHV5 infection. This fits with previous data that show that while loggerheads are infrequently observed with FP tumors, the disease is often relatively mild compared to the severe, sometimes fatal tumor syndrome seen in green turtles with ChHV5 infections [7,54].

Here, we report 10 instances in which a turtle tested positive for either ChHV5 DNA or antibodies to ChHV5, or both, and subsequently tested negative while undergoing rehabilitation. This suggests that as turtles were rehabilitated and their cause(s) of stranding resolved, their underlying, subclinical ChHV5 infections became more quiescent and undetectable by the assays used here. This observation fits with our general knowledge of herpesvirus biology, in which a herpesvirus may reactivate or recrudesce with comorbid conditions such as stress, coinfections, or injury [52,55,56]. Likewise, there were five instances in which turtles initially tested negative for ChHV5 via qPCR or ELISA, and subsequently tested positive. Again, this finding is likely related to the immune status of the turtles at the time the samples were collected [46,47] or to exposure in rehabilitation. Although there were not any significant differences in the PCV or plasma total solids concentrations between turtles that did or did not test positive for ChHV5 via qPCR or ELISA, PCV and total solid measurements alone do not fully encompass the differences in the immune function of sea turtles. Future studies could provide more insight on this issue by testing turtles without FP for ChHV5 DNA and antibodies in conjunction with more in-depth immune function tests such as oxidative burst and phagocytosis by flow cytometry [57]. Plasma protein electrophoresis may also provide relevant data on immune system activity [58].

## 5. Conclusions

The results of this study provide information on current and prior ChHV5 infections in two sea turtle species that are vulnerable to the development of FP tumors, in both wild and captive settings, in North Carolina, USA. Positive results from the qPCR assay used here are interpreted as an active ChHV5 infection due to the presence of circulating viral DNA. The lack of detectable ChHV5 DNA in presumably healthy, free-ranging sea turtles is contrasted with the finding of ChHV5 DNA-emia in sick and recovering turtles in rehabilitative care. This suggests that turtles with injuries, cold stunning, chronic debilitation, and other health problems that lead to stranding may have a recrudescence of previous viral infections (likely associated with immunosuppression) or may be subject to new ChHV5 infections in captive care. Several of these cases of subclinical viral DNA-emia were shown to resolve over time with supportive care and the resolution of comorbid conditions, concurrent with an overall improvement of health and nutritional conditions. Both green and loggerhead turtles were significantly more likely to test positive for ChHV5 antibodies using ELISA than for ChHV5 DNA using qPCR. Since herpesvirus infections are generally thought to be lifelong, positive ELISA results indicating the presence of circulating antibodies are interpreted as a prior or latent ChHV5 infection (rather than a prior ‘exposure’). Because qPCR and antibody detection assays give different types of diagnostic information on ChHV5 infection, using the two tests in tandem gives a more complete picture of the infection status than using either assay alone. Although the prevalence of FP is thought to be relatively low in the northwestern Atlantic sea turtle populations including North Carolina, the data presented here demonstrate that ChHV5 is still present within the green and loggerhead sea turtle populations inhabiting this region.

## Figures and Tables

**Table 1:** Number of sea turtles sampled for this study, according to species and circumstances of encounter.

Type of Turtle Encounter	Green Turtles	Loggerheads	Total
Free-ranging	20	26	46
Rehabilitating–single samples	52	65	117
Rehabilitating–paired samples	41	21	62
Total	113	112	225
Number of samples in rehabilitating turtles			
2	23	6	29
3	14	8	22
4	4	4	8
5	0	1	1
6	0	1	1
7	0	1	1
Total	41	21	62

**Table 2:** Qualitative results for the samples tested using quantitative polymerase chain reaction (qPCR) to detect chelonid alphaherpesvirus 5 (ChHV5) DNA and using an enzyme-linked immunosorbent assay (ELISA) to detect antibodies to ChHV5.

Type of Turtle Encounter	Number (%) of Samples Positive for ChHV5 DNA via qPCR	Number (%) of Samples Positive for Antibodies to ChHV5 via ELISA	Number (%) of Samples That Tested Positive via both Assays	Total Number (%) Positive for ChHV5
Green turtles (*Chelonia mydas*)				
Free-ranging	0/20 (0%)	N/A	N/A	0/20 (0%)
Rehabilitating–single samples	7/52 (13.5%)	0/9 (0%)	0/9 (0%)	7/52 (13.5%)
Rehabilitating–paired samples	1/104 (1.0%)	9/26 (34.6%)	0/26 (0%)	10/104 (9.6%)
Rehabilitating–individual turtles with paired samples	1/41 (2.4%)	6/16 (37.5%)	0/16 (0%)	7/41 (17.1%)
Total number of samples	8/176 (4.6%)	9/35 (25.7%)	0/35 (0%)	17/176 (9.7%)
Total number of turtles	8/113 (7.1%)	6/25 (24.0%)	0/16 (0%)	14/113 (12.4%)
Loggerheads (*Caretta caretta*)				
Free-ranging	0/26 (0%)	N/A	N/A	0/26 (0%)
Rehabilitating–single samples	5/65 (7.7%)	9/13 (69.2%)	1/13 (7.7%)	13/65 (20.3%)
Rehabilitating–paired samples	5/71 (7.0%)	35/46 (76.1%)	2/46 (4.3%)	38/71 (53.5%)
Rehabilitating–individual turtles with paired samples	5/21 (23.8%)	17/18 (94.4%)	2/18 (11.1%)	18/21 (85.7%)
Total number of samples	10/162 (6.2%)	44/59 (74.6%)	3/59 (5.1%)	51/162 (31.5%)
Total number of turtles	10/112 (8.9%)	26/31 (83.9%)	3/31 (9.7%)	31/112 (27.7%)
Total number of samples–Both species	18/338 (5.3%)	53/94 (56.4%)	3/94 (3.2%)	68/338 (20.1%)
Total number of turtles–Both species	18/225 (8.0%)	32/56 (55.4%)	3/47 (6.4%)	45/225 (6.2%)

**Table 3:** Results of N–1 chi-squared tests to analyze differences in the proportional data resulting from the two diagnostic tests included in the study, quantitative polymerase chain reaction (qPCR) and enzyme-linked immunosorbent assay (ELISA). CI, confidence interval; df, degrees of freedom; * denotes statistically significant differences in proportions with α = 0.05; ^a^ Green turtles, loggerheads, and both species combined were more likely to test positive for ChHV5 via ELISA than via qPCR; ^b^ Loggerheads were more likely than green turtles to test positive for ChHV5 via ELISA; ^c^ Loggerheads were more likely than green turtles to test positive for ChHV5 via both assays combined.

	Percent Difference (%)	95% CI	χ^2^	df	*p*
Tested positive for ChHV5 via qPCR versus ELISA					
Green turtles ^a^	21.1	8.8–37.6%	17.4	1	<0.001 *
Loggerheads ^a^	68.4	55.1–78.2%	109.0	1	<0.001 *
Both species ^a^	51.1	40.6–60.9%	139.6	1	<0.001 *
qPCR results for free-ranging versus rehabilitating turtles					
Green turtles	5.1	–11.2–9.8%	1.1	1	0.30
Loggerheads	7.4	–5.9–13.0%	2.0	1	0.15
Both species	6.2	–1.9–9.5%	3.0	1	0.08
Tested positive for ChHV5 via qPCR upon entry into rehabilitation versus established patients					
Green turtles	8.7	–1.0–16.2%	3.7	1	0.06
Loggerheads	12.9	–1.9–35.6%	2.7	1	0.10
Both species	1.7	–8.3–8.2%	0.2	1	0.68
Tested positive for ChHV5 via ELISA upon entry into rehabilitation versus established patients					
Green turtles	6.7	–3.7–29.9%	2.7	1	0.10
Loggerheads	3.3	–19.9–30.4%	0.1	1	0.80
Both species	6.9	–16.5–28.8%	0.3	1	0.58
Green turtles versus loggerheads that tested positive for ChHV5 via qPCR	1.6	–3.4–6.9%	0.4	1	0.52
Green turtles versus loggerheads that tested positive for ChHV5 via ELISA ^b^	48.9	28.4–63.8%	21.1	1	<0.001 *
Green turtles versus loggerheads that tested positive for ChHV5 via both assays ^c^	21.8	13.3–30.1%	24.8	1	<0.001 *

**Table 4:** Results of the statistical analysis of the level of agreement between the two diagnostic tests for chelonid alphaherpesvirus 5 (quantitative polymerase chain reaction and enzyme linked immunosorbent assay) that were applied to samples from green (*Chelonia mydas*) and loggerhead (*Caretta caretta*) sea turtles. CI, confidence interval; SE, standard error.

Species	N	Kappa Statistic (κ)	SE of κ	95% CI	Interpretation
Loggerheads	60	0.03	0.02	–0.01–0.08	Fair agreement
Green turtles	38	–0.05	0.05	–0.15–0.05	No agreement

**Table 5:** Summarized quantitative data on the copy number (per µg DNA) for 18 rehabilitating sea turtles that tested positive for chelonid alphaherpesvirus 5 (ChHV5) UL30 DNA via quantitative polymerase chain reaction (qPCR) and sequencing. SD, standard deviation.

Species	N	ChHV5 UL30 DNA Copy Number	
		Average ± SD	Range
Loggerheads (*Caretta caretta*)	10	1179 ± 2479	50–8067
Green turtles (*Chelonia mydas*)	8	98 ± 88	50–304
Total (both species)	18	698 ± 1888	50–8067
